# Job Strain, Worksite Support, and Nutrient Intake among Employed Japanese Men and Women

**DOI:** 10.2188/jea.16.79

**Published:** 2006-03-14

**Authors:** Norito Kawakami, Akizumi Tsutsumi, Takashi Haratani, Fumio Kobayashi, Masao Ishizaki, Takeshi Hayashi, Osamu Fujita, Yoshiharu Aizawa, Shogo Miyazaki, Hisanori Hiro, Takeshi Masumoto, Shuji Hashimoto, Shunichi Araki

**Affiliations:** 1 Hygiene and Preventive Medicine, Okayama University Graduate School of Medicine, Dentistry & Pharmaceutical Sciences.; 2 National Institute of Industrial Health.; 3 Aichi Medical University.; 4 Kanazawa Medical University.; 5 HITACHI Information & Telecomunication Systems Shinkawasaki Health Care Center.; 6 Kariya General Hospital Eastern Division.; 7 Kitasato University School of Medicine.; 8 Meiji University Law School.; 9 Adecco Health Support Center.; 10 Mitsubishi Chemical Corporation; 11 Fujita Health University School of Medicine.

**Keywords:** Coronary Disease, Risk Factors, Dietary Fats, Stress, Social Support, Japan

## Abstract

**BACKGROUND:**

The association of job strain (as defined by the job demands/control model) and worksite support with nutrient intake is not clear.

**METHODS:**

A questionnaire survey was conducted of 25,104 workers employed in nine companies in Japan. Job strain and worksite support were assessed using the Job Content Questionnaire. Daily intake of 17 nutrients was measured using a dietary history questionnaire. Data from 15,295 men and 2,853 women were analyzed, controlling for age, education, marital status, occupation, and study site.

**RESULTS:**

Among men, job strain was positively associated with average daily intakes of fat, vitamin E, cholesterol, poly- and mono-unsaturated fatty acids (p for trend<0.05), and worksite support was positively associated with average daily intakes of total energy, crude fiber, retinol, carotene, vitamins A, C, and E, cholesterol, and saturated fatty acid (p for trend<0.05). Among women, worksite support was positively associated with average daily intakes of total energy, protein, vitamin E, and polyunsaturated fatty acid (p for trend<0.05). However these differences were generally small.

**CONCLUSIONS:**

The present study showed that job strain and worksite support were only weakly and inconsistently associated with nutritional intakes. It does not seem that changes in nutritional intakes explain the association between job strain or worksite support and coronary heart disease.

Psychological job strain, which is defined as the combination of greater psychological job demands and lower job control in the job strain model,^1^^,^^2^ has been linked to coronary heart disease (CHD). It has been also associated with several CHD risk factors, such as high blood pressure and, to a lesser extent, serum total cholesterol.^2^^,^^3^ Job strain has been found to be associated with elevated blood pressure in a non-Western country, i.e., Japan, as well.^4^

Changes in diet, in addition to smoking and drinking, are possible mediating behavior variables linking job strain to CHD.^3^ Thus far, only a few studies have been conducted to clarify the association between job strain and diet. Hellerstedt and Jeffery found that men who reported high job strain had significantly greater calorie intake from high-fat food items.^5^ A consistent pattern was also observed in a recent study of a Japanese rural population: job strain was significantly and positively associated with meat consumption among men with non-agricultural occupations.^6^ The same study also indicated that vegetable consumption was lower in workers with high job strain, while another study found that job strain was not significantly associated with fruit or vegetable consumption.^7^

These previous studies^5^^-^^7^ examined only gross food consumption and associations with job strain, and none used a dietary history questionnaire to measure nutrient intake. While fat consumption was associated with CHD risk, saturated fat has been recognized as a major factor for CHD,^8^ and other types of fat, such as poly- and monounsaturated fatty acids, have not been associated with CHD and may in fact even provide protection against CHD risk.^8^^,^^9^ Cholesterol and salt are also important dietary elements that have been associated with CHD risk.^8^ Some studies have reported that protein, fiber, and several antioxidants (carotene, vitamins C and E) were protective against CHD.^9^ The question of whether job strain is associated with the consumption of fat components, cholesterol, salt, and some other micronutrients such as vitamins should be examined, and further research on food items such as high-fat foods, vegetables, and fruits should be conducted in order to clarify the mediating role in the link between job strain and CHD.

Epidemiologic studies have also suggested that worksite support (i.e., social support at work) is inversely associated with CHD, either as a main effect or a buffering effect against job strain,^10^^-^^12^ although there was a study that failed to show such an effect.^13^ Other studies have reported that low worksite support was associated with increased blood pressure^14^^,^^15^ and hyperlipidemia^16^ although these findings are somewhat conflicting. Differences in diet may be responsible for the link between worksite support and CHD. To date, only one study has examined the relationship, and it found no significant association between worksite support (or its interaction with job strain) and the consumption of vegetables or fruit,^7^ while social interaction in general has been positively associated with vegetable and fruit intake.^17^^,^^18^

In order to examine the possible mediating role of dietary differences linking job strain and worksite support to CHD, in this study we analyzed data from a baseline survey of a large-scale prospective cohort study of employed men and women in Japan, called the Japan Work Stress and Health Cohort (JSTRESS) Study. More specifically, we investigated the association of job strain and worksite support with the consumption of 17 selected nutrients, including fat components, cholesterol, salt and vitamins, while controlling for other relevant factors.

## METHODS

### Subjects

A total of nine companies and factories located in the Kanto (East Japan) and Chubu (Central Japan) areas of Japan were selected as study sites of the JSTRESS Study.^19^ These sites were a light-metal factory, three electrical factories, two steel factories, a heavy-metal factory, a manufacturing factory, and an automobile factory. A total of 29,471 employees were invited to participate in the study, and a written consent was obtained. Recruitment strategies were slightly different among study sites. All employees at four study sites were invited to participate in the study, while at three other study sites employees who attended a compulsory health checkup over a certain period were invited to participate. At another study site, only men who attended a compulsory health checkup were invited to participate. Finally, at one study site, all supervisors and managers were invited to participate. The surveys were conducted from April 1996 through May 1998. A total of 25,104 questionnaires were returned, and the response rate ranged from 43% to 100% at the study sites, with the average being 85%. We excluded 3,026 responses from one study site that were collected during the health checkups that took place between June and November 1997 (80% of the respondents from the site) because the questionnaire distributed during that period lacked job stressor scales due to an editorial mistake. Furthermore, 3,739 participants were excluded (17% of the eligible 22,078 respondents) because one or more of the items in the questionnaire had not been answered. In total, therefore, data from 18,148 respondents (15,295 men and 2,853 women) were analyzed in the final analysis.

### Assessment of Job Strain and Worksite Support

Job strain and worksite support were measured by using the scales of the Job Content Questionnaire (JCQ): job demands, job control, and worksite social support.^20^^,^^21^ The Japanese version of the scales had shown acceptable levels of reliability and validity in a previous study.^22^ Cronbach’s alpha reliability coefficients for the scales ranged from 0.61 to 0.89 for men and from 0.65 to 0.87 for women. In addition, the scales showed factor-based validity, with distributions of the scale scores across age groups and occupations being in an expected direction.^22^ Job strain ratio was calculated as a ratio of job demands to job control. The ratio was multiplied by 2 to adjust for the difference in scoring ranges between the job demand scale (12-48) and the job control scale (24-96). This job strain ratio has been used in seven previous studies that yielded significant associations of job strain with myocardial infarction and CHD risk factors.^3^^,^^23^ We used a total score of worksite social support by adding the scores of supervisor support and coworker support, as well as each of the two above scale scores because they were closely correlated (r=0.44). The correlation between the job strain ratio and the worksite social support score was moderate (r=-0.26 for men and r=-0.24 for women). Distributions of the JCQ scores are shown by gender in Table 1. The subjects were classified into four quartiles (lowest, second-lowest, second-highest, and highest) based on a gender-specific distribution of job strain and worksite support (see Appendix).

**Table 1.** Averages and standard deviations (SDs) (or Q1 and Q3 values) of age, estimated nutrient intake, and job stressor variables among men and women: Baseline data of The Japan Work Stress and Health Cohort Study (1996-1998).

	Men (n=15,295)				Women (n=2,853)			
	
	Mean	SD	Q1	Q3	Mean	SD	Q1	Q3
Age (year)	40.8	8.8			36.3	10.5		
	Nutrient intake*							
Total energy (kcal)	2242		1911		2008		1700	2376
Protein (g)	84.2		79.5	2613	89.6		85.2	94.1
Fat (g)	57.1		52.3	89.0	61.9		57.4	66.0
Carbohydrate (g)	315.7		304.6	62.1	316.5		306.3	328.8
Crude fiber (g)	4.56		3.95	331.0	5.02		4.35	5.67
Calcium (mg)	604.5		514.2	5.15	728.0		625.3	843.2
Retinol (*μ*g)	369.2		195.1	702.5	299.4		202.4	390.7
Carotene (*μ*g)	3855.3		3001.1	596.1	4686.3		3660.7	5943.3
VA (IU)	3257.9		2538.9	4848.6	3467.4		2741.2	4291.6
VC (mg)	134.2		105.0	4134.1	152.4		121.1	189.9
VD (IU)	88.4		69.2	169.3	99.8		77.5	126.4
VE (mg)	7.66		6.96	109.8	8.48		7.76	9.19
Salt (g)	12.5		11.5	8.38	13.9		12.8	15.0
Cholesterol (mg)	290		246	13.5	310		268	354
Saturated fatty acid (g)	12.4		11.1	339	13.3		11.8	14.6
Polyunsaturated fatty acid (g)	13.4		12.4	13.7	14.1		13.0	15.2
Monounsaturated fatty acid (g)	16.8		15.1	14.5	17.8		16.3	19.2
				18.4				
	Job stressor score:							
Job demands	32.8	5.2			31.5	5.2		
Job control	67.5	10.9			59.0	10.4		
Job strain^†^	1.00	0.24			1.11	0.32		
Supervisor support	10.8	2.2			10.5	2.4		
Coworker support	11.2	1.6			11.0	1.7		
Worksite support	22.0	3.2			21.6	3.4		

* : Nutrient intake (excluding total energy) was adjusted for total energy after logarithm transformation of crude values by the method of Willett (1990). Geometric means, Q1 (lower 25%), and Q3 (upper 25%) values were shown for nutrient intake.

†: Job strain = job demands / job control × 2

### Nutrient Intake

In this study, nutrient intake was assessed using a modified version of a 31-item dietary history questionnaire (DHQ).^24^ The original DHQ was developed by shortening a quantitative food frequency questionnaire used for cancer epidemiology in Japan^25^ and designed to estimate the daily intake of 17 macro and micronutrients in a Japanese population according to the 4th edition of the Standard Tables of Food Composition in Japan^26^ (see Table 1). The original DHQ^24^ consisted of questions about the average frequency of consumption and the average portion size of 31 selected food items during the past year. Questions concerning portion size were deleted in this study in order to make the task easier for respondents. In place of these questions, a gender-specific average portion for each item was used so that nutrient intake could be estimated. This modified version of the DHQ still had moderate to high validity when compared with estimated nutritional intakes based on 12 one-day dietary records over one-year period among 37 volunteers (Takatsuka, Shimizu, Kawakami, et al., unpublished data). Spearman’s partial correlation coefficients (r_s_) adjusted for gender between the DHQ and the three-day records were high (r_s_=0.40-0.59) for crude fiber, calcium, vitamins C, D, and E, and salt; moderate (r_s_=0.25-0.39) for total energy, protein, carbohydrates, calcium, carotene, cholesterol, and saturated and polyunsaturated fatty acid; and relatively low (r_s_=0.15-0.24) for retinol, vitamin A, and monounsaturated fatty acid (Takatsuka, Shimizu, Kawakami, et al., unpublished data). Comparisons of the estimated average values with those from the three-day records indicated that the DHQ overestimated vitamin C (+112%), retinol (+58%), carotene (+52%), and crude fiber (+16%). Otherwise, differences in estimated averages between the DHQ and the three-day records were within 10% (Takatsuka, Shimizu, Kawakami, et al., unpublished data).

After logarithmic transformation, nutrient intake was adjusted for total energy intake by using the method proposed by Willett^27^ and applying a linear regression method. The distributions of nutrient intakes by gender are shown in Table 1.

### Other Covariates

Other covariates included age, education, marital status, and occupation, which are possible determinants of job strain, worksite support,^2^ or nutrition intake.^28^ The questionnaire was used to collect this information. Three age categories were used: 18-34, 35-44, and 45-60 years old. Nine occupational categories, defined according to the International Standardized Classification of Occupations 1988, were used: managers, professionals, technicians, clerks, service and sales workers, craft and other skilled workers, machine operators and assemblers, and laborers. Two categories were used for education level: 12 years or less and 13 years or more. Two categories were used for marital status: married and not married (single, divorced, or widowed). In addition, eight dummy variables were designed to adjust for the differences among the nine study sites in a multivariate analysis. A chronic condition was defined as one receiving medical treatment for any of the following conditions: hypertension, diabetes, hyperlipidemia, cancer, CHD, and stroke. A total of 657 (4.3%) men and 60 (2.1%) women had a chronic condition.

### Statistical Analysis

The 17 dietary nutrient intakes (log transformed values) were compared among the quartiles of job strain and worksite support. The averages and their 95% confidence intervals were calculated based on the distribution of the log-transformed values and are shown in Tables after anti-log transformation. Statistical differences among the quartiles of job strain and worksite support were tested using an analysis of covariance (ANCOVA) controlling for age. The linear trend was also tested controlling for age.

In order to examine the unique association of job strain and worksite support to nutrient intakes, we conducted an ANCOVA for each of the 17 nutrient intakes on the quartiles of job strain (or job demands and job control) or worksite social support, controlling for age group, education, marital status, occupation category, and study sites. The significance for a linear trend between job strain or worksite support and nutritional intake was tested by assigning consecutive numbers (1-4) to the quartiles. Similar ANCOVAs were conducted for those who did not report a chronic condition (14,638 men and 2,793 women) in order to confirm findings among a “healthy” population. These statistical analyses were conducted using SAS^®^ package version 6.14 on a PC.^29^

## RESULTS

### Job Strain and Nutrient Intake

Among men (Table 2, top), job strain was significantly and positively associated with fat, salt, and poly- and monounsaturated-fatty acid intakes, while it was significantly and negatively associated with intakes of carbohydrate, crude fiber, calcium, carotene, and vitamin C. Among women (Table 2, bottom), job strain was significantly and positively associated with retinol intake.

**Table 2.** Average daily intakes of 17 nutrients* by the quartiles of job strain among employed men and women in Japan: Baseline data of The Japan Work Stress and Health Cohort (JSTRESS) Study (1996-1998).

	Job strain^†^								P for trend^‡^

	1=low		2		3		4=high		
			
	Mean	(95% CI)	Mean	(95% CI)	Mean	(95% CI)	Mean	(95% CI)	

	Men								
n	3794		3838		3770		3893		
Total energy (kcal)	2243	(2226 - 2261)	2242	(2224 - 2260)	2238	(2220 - 2256)	2244	(2225 - 2264)	0.774
Protein (g)	84.1	(83.9 - 84.4)	84.3	(84.1 - 84.5)	84.1	(83.8 - 84.3)	84.2	(83.9 - 84.4)	0.270
Fat (g)	56.6	(56.3 - 56.8)	57.1	(56.9 - 57.4)	57.2	(57.0 - 57.5)	57.6	(57.4 - 57.9)	<0.001
Carbohydrate (g)	316.4	(315.7 - 317.1)	315.6	(314.9 - 316.3)	315.5	(314.7 - 316.2)	315.4	(314.7 - 316.2)	0.009
Crude fiber (g)	4.59	(4.56 - 4.62)	4.59	(4.56 - 4.62)	4.54	(4.52 - 4.57)	4.51	(4.48 - 4.54)	<0.001
Calcium (mg)	609.6	(605.2 - 614.0)	607.6	(603.2 - 612.0)	602.6	(598.3 - 607.0)	598.5	(594.2 - 603.0)	0.007
Retinol (*μ*g)	362.1	(354.1 - 370.2)	367.1	(359.1 - 375.3)	374.8	(366.3 - 383.4)	372.8	(364.1 - 381.7)	0.240
Carotene (*μ*g)	3918.7	(3874.2 - 3963.8)	3904.7	(3860.5 - 3949.3)	3843.0	(3799.2 - 3887.2)	3758.8	(3715.6 - 3802.5)	<0.001
VA (IU)	3261.4	(3224.0 - 3299.1)	3272.5	(3235.7 - 3309.8)	3267.4	(3229.7 - 3305.5)	3231.0	(3191.5 - 3270.9)	0.061
VC (mg)	135.9	(134.3 - 137.4)	135.5	(134.0 - 137.1)	133.5	(132.0 - 135.0)	131.9	(130.3 - 133.5)	<0.001
VD (IU)	89.1	(88.1 - 90.1)	89.1	(88.2 - 90.1)	88.2	(87.2 - 89.2)	87.1	(86.1 - 88.1)	0.405
VE (mg)	7.65	(7.61 - 7.68)	7.68	(7.65 - 7.72)	7.66	(7.62 - 7.69)	7.65	(7.62 - 7.69)	0.979
Salt (g)	12.4	(12.4 - 12.5)	12.5	(12.5 - 12.5)	12.5	(12.4 - 12.5)	12.5	(12.4 - 12.5)	0.002
Cholesterol (mg)	288	(286 - 291)	290	(288 - 292)	290	(288 - 292)	291	(289 - 293)	0.142
Saturated fatty acid (g)	12.3	(12.3 - 12.4)	12.4	(12.3 - 12.4)	12.4	(12.3 - 12.4)	12.4	(12.3 - 12.5)	0.773
Polyunsaturated fatty acid (g)	13.4	(13.3 - 13.5)	13.4	(13.4 - 13.5)	13.4	(13.4 - 13.5)	13.5	(13.5 - 13.6)	0.004
Monounsaturated fatty acid (g)	16.6	(16.5 - 16.7)	16.8	(16.7 - 16.8)	16.8	(16.7 - 16.9)	16.9	(16.8 - 17.0)	0.002

	Women								
n	704		708		727		714		
Total energy (kcal)	1980	(1941 - 2020)	2040	(1999 - 2081)	2003	(1965 - 2041)	2009	(1968 - 2051)	0.678
Protein (g)	89.4	(88.9 - 89.9)	89.4	(88.9 - 89.9)	90.1	(89.6 - 90.6)	89.4	(88.9 - 90.0)	0.945
Fat (g)	61.8	(61.3 - 62.4)	61.9	(61.4 - 62.5)	62.0	(61.5 - 62.5)	61.6	(61.1 - 62.2)	0.953
Carbohydrate (g)	317.5	(316.1 - 318.8)	316.2	(314.9 - 317.6)	316.0	(314.6 - 317.3)	316.4	(314.9 - 317.9)	0.342
Crude fiber (g)	5.07	(5.00 - 5.15)	4.99	(4.91 - 5.06)	5.07	(4.99 - 5.15)	4.97	(4.90 - 5.05)	0.160
Calcium (mg)	729.2	(717.8 - 740.7)	723.6	(712.1 - 735.3)	735.7	(724.0 - 747.5)	723.3	(711.7 - 735.1)	0.550
Retinol (*μ*g)	292.9	(282.3 - 304.0)	302.1	(289.9 - 314.8)	292.5	(281.0 - 304.4)	310.4	(297.5 - 323.9)	0.042
Carotene (*μ*g)	4778.6	(4653.1 - 4907.4)	4627.0	(4504.4 - 4752.9)	4774.7	(4649.1 - 4903.7)	4567.9	(4449.5 - 4689.4)	0.091
VA (IU)	3484.3	(3402.1 - 3568.5)	3442.3	(3358.8 - 3527.9)	3494.7	(3407.3 - 3584.4)	3448.1	(3363.4 - 3534.9)	0.989
VC (mg)	155.5	(151.6 - 159.4)	151.0	(147.4 - 154.8)	153.3	(149.5 - 157.2)	149.9	(146.1 - 153.8)	0.092
VD (IU)	97.2	(94.6 - 99.9)	99.1	(96.4 - 101.8)	103.2	(100.5 - 106.0)	99.5	(96.7 - 102.5)	0.569
VE (mg)	8.50	(8.42 - 8.59)	8.45	(8.37 - 8.53)	8.53	(8.45 - 8.61)	8.45	(8.37 - 8.54)	0.616
Salt (g)	13.8	(13.6 - 13.9)	13.9	(13.7 - 14.0)	14.1	(13.9 - 14.2)	14.0	(13.8 - 14.1)	0.069
Cholesterol (mg)	311	(306 - 316)	306	(301 - 311)	312	(307 - 317)	310	(305 - 315)	0.555
Saturated fatty acid (g)	13.3	(13.2 - 13.5)	13.2	(13.1 - 13.4)	13.2	(13.1 - 13.4)	13.2	(13.1 - 13.4)	0.541
Polyunsaturated fatty acid (g)	14.1	(13.9 - 14.2)	14.1	(14.0 - 14.3)	14.2	(14.1 - 14.3)	14.1	(14.0 - 14.2)	0.630
Monounsaturated fatty acid (g)	17.9	(17.7 - 18.1)	17.8	(17.7 - 18.0)	17.8	(17.6 - 18.0)	17.8	(17.6 - 18.0)	0.926

* : Nutrient intake (excluding total energy) was adjusted for total energy after logarithm transformation of crude values by the method of Willett (1990).

Geometric mean and 95% confidence intervals were estimated on the distribution of log transformed value.

†: Job strain was classifed into quartiles. P for diff.: P for difference (d.f=3).

‡: Adjusted for age.

### Worksite Support and Nutrient Intake

Among men (Table 3, top), worksite support was significantly and positively associated with total energy and intakes of crude fiber, calcium, retinol, carotene, vitamins A, C, and E, and saturated fatty acid. Among women (Table 3, bottom), worksite support was significantly and positively associated with total energy and intakes of protein, crude fiber, carotene, vitamins C and E, and polyunsaturated fatty acid.

**Table 3.** Average daily intakes of 17 nutrients* by the quartile of worksite support among employed men and women in Japan: Baseline data of The Japan Work Stress and Health Cohort (JSTRESS) Study (1996-1998).

	Worksite Support^†^								P for trend^‡^

	1=low		2		3		4=high		
			
	Mean	(95% CI)	Mean	(95% CI)	Mean	(95% CI)	Mean	(95% CI)	

	Men								
n	2881		4309		2682		5423		
Total energy (kcal)	2219	(2197 - 2240)	2232	(2216 - 2249)	2225	(2205 - 2246)	2270	(2255 - 2286)	<0.001
Protein (g)	84.2	(83.9 - 84.5)	84.0	(83.8 - 84.2)	83.9	(83.6 - 84.2)	84.4	(84.2 - 84.6)	0.149
Fat (g)	57.2	(56.9 - 57.6)	57.0	(56.8 - 57.3)	57.1	(56.8 - 57.3)	57.2	(57.0 - 57.4)	0.689
Carbohydrate (g)	315.0	(314.1 - 315.9)	316.0	(315.3 - 316.7)	316.0	(315.2 - 316.8)	315.8	(315.2 - 316.4)	0.344
Crude fiber (g)	4.52	(4.49 - 4.56)	4.53	(4.50 - 4.56)	4.53	(4.50 - 4.57)	4.61	(4.59 - 4.64)	<0.001
Calcium (mg)	600.2	(595.1 - 605.4)	603.9	(599.8 - 608.0)	599.1	(594.1 - 604.1)	610.1	(606.4 - 613.8)	0.006
Retinol (*μ*g)	367.5	(357.7 - 377.7)	365.1	(357.5 - 372.9)	358.8	(349.3 - 368.5)	378.6	(371.4 - 385.8)	0.048
Carotene (*μ*g)	3772.3	(3720.9 - 3824.5)	3839.6	(3798.3 - 3881.3)	3825.6	(3775.0 - 3876.9)	3927.8	(3890.6 - 3965.4)	<0.001
VA (IU)	3227.0	(3181.5 - 3273.2)	3234.0	(3199.0 - 3269.4)	3195.9	(3152.7 - 3239.7)	3325.1	(3292.9 - 3357.5)	<0.001
VC (mg)	131.7	(129.9 - 133.6)	132.6	(131.1 - 134.0)	133.3	(131.5 - 135.1)	137.2	(135.9 - 138.6)	<0.001
VD (IU)	88.8	(87.6 - 90.1)	87.8	(86.8 - 88.7)	88.0	(86.8 - 89.1)	88.8	(88.0 - 89.6)	0.900
VE (mg)	7.66	(7.62 - 7.70)	7.62	(7.59 - 7.65)	7.64	(7.60 - 7.68)	7.70	(7.68 - 7.73)	0.011
Salt (g)	12.5	(12.4 - 12.6)	12.4	(12.4 - 12.5)	12.4	(12.4 - 12.5)	12.5	(12.4 - 12.5)	0.701
Cholesterol (mg)	290	(287 - 293)	288	(286 - 290)	290	(287 - 292)	291	(289 - 293)	0.087
Saturated fatty acid (g)	12.3	(12.2 - 12.4)	12.4	(12.3 - 12.4)	12.3	(12.2 - 12.4)	12.4	(12.4 - 12.5)	0.017
Polyunsaturated fatty acid (g)	13.5	(13.4 - 13.5)	13.4	(13.4 - 13.5)	13.5	(13.4 - 13.5)	13.4	(13.4 - 13.5)	0.541
Monounsaturated fatty acid (g)	16.8	(16.7 - 16.9)	16.7	(16.6 - 16.8)	16.7	(16.6 - 16.8)	16.8	(16.7 - 16.9)	0.486

	Women								
n	684		507		857		805		
Total energy (kcal)	1988	(1947 - 2029)	1974	(1929 - 2020)	1990	(1955 - 2027)	2066	(2026 - 2106)	0.001
Protein (g)	89.1	(88.6 - 89.7)	89.8	(89.1 - 90.4)	89.4	(88.9 - 89.9)	90.1	(89.6 - 90.6)	0.030
Fat (g)	61.8	(61.2 - 62.3)	62.2	(61.6 - 62.9)	61.8	(61.4 - 62.3)	61.7	(61.2 - 62.2)	0.730
Carbohydrate (g)	316.4	(315.0 - 317.9)	315.5	(313.7 - 317.3)	316.9	(315.7 - 318.2)	316.8	(315.5 - 318.1)	0.537
Crude fiber (g)	4.95	(4.88 - 5.03)	5.05	(4.96 - 5.14)	4.99	(4.92 - 5.06)	5.11	(5.03 - 5.19)	0.015
Calcium (mg)	718.4	(705.9 - 731.1)	740.8	(726.9 - 755.0)	716.7	(706.8 - 726.7)	740.4	(729.6 - 751.3)	0.081
Retinol (*μ*g)	299.8	(287.7 - 312.5)	306.4	(290.9 - 322.8)	294.6	(284.3 - 305.2)	299.7	(288.9 - 311.0)	0.531
Carotene (*μ*g)	4545.5	(4419.5 - 4675.0)	4758.0	(4614.1 - 4906.4)	4629.4	(4521.5 - 4740.0)	4826.1	(4705.3 - 4950.0)	0.009
VA (IU)	3387.0	(3299.4 - 3476.9)	3544.5	(3440.0 - 3652.2)	3416.4	(3342.4 - 3492.0)	3544.0	(3464.7 - 3625.1)	0.076
VC (mg)	149.2	(145.4 - 153.1)	154.1	(149.5 - 158.8)	150.4	(147.0 - 153.9)	156.3	(152.7 - 160.0)	0.038
VD (IU)	99.4	(96.5 - 102.3)	98.6	(95.3 - 102.0)	100.3	(97.9 - 102.7)	100.3	(97.7 - 102.9)	0.357
VE (mg)	8.41	(8.33 - 8.49)	8.46	(8.37 - 8.56)	8.48	(8.40 - 8.55)	8.57	(8.49 - 8.65)	0.007
Salt (g)	13.9	(13.8 - 14.0)	13.9	(13.8 - 14.1)	13.9	(13.8 - 14.1)	13.9	(13.8 - 14.0)	0.878
Cholesterol (mg)	306	(301 - 311)	309	(303 - 315)	311	(307 - 316)	311	(307 - 316)	0.084
Saturated fatty acid (g)	13.2	(13.0 - 13.3)	13.4	(13.2 - 13.6)	13.1	(13.0 - 13.3)	13.3	(13.2 - 13.5)	0.781
Polyunsaturated fatty acid (g)	14.0	(13.9 - 14.2)	14.1	(13.9 - 14.2)	14.2	(14.1 - 14.3)	14.2	(14.1 - 14.3)	0.042
Monounsaturated fatty acid (g)	17.8	(17.6 - 17.9)	17.9	(17.7 - 18.2)	17.8	(17.7 - 18.0)	17.8	(17.7 - 18.0)	0.799

* : Nutrient intake (excluding total energy) was adjusted for total energy after logarithm transformation of crude values by the method of Willett (1990). Geometric mean and 95% confidence intervals were estimated on the distribution of log transformed value.

†: Worksite support was classifed into quartiles. P for diff.: P for difference (d.f=3).

‡: Adjusted for age.

### Multivariate Analysis

Among men (Table 4, top), job strain was significantly and positively associated with intakes of fat, vitamin E, cholesterol, and poly- and monounsaturated fatty acid after controlling for other covariates, including worksite support; however, the differences were generally small (0.7 g/day, 0.06 mg/day, 3 mg/day, 0.1 g/day and 0.2 g/day, respectively, between the highest and lowest quartiles). Worksite support was significantly and positively associated with total energy and intakes of crude fiber, retinol, carotene, vitamins A, C and E, cholesterol, and saturated fatty acid; however, the differences were again small (50 kcal/day, 0.05 g/day, 11.5 *μ*g /day, 91.4*μ*g /day, 70.7 IU/day, 3.8 mg/day, 0.03 mg/day, 2 mg/day, and 0.1 g/day, respectively, differences between the highest and lowest quartiles). Among women (Table 4, bottom), job strain was not significantly associated with any nutrient, after controlling for other covariates. Worksite support was significantly and positively associated with total energy and intakes of protein, vitamin E, and polyunsaturated fatty acid; the differences tended to be small (92 kcal/day, 1.0 g/day, 0.2 mg/day, and 0.2 g/day, respectively, between the highest and lowest quartiles). Moreover, for both men and women, when an interaction term was added to the model (df=9) between job strain and worksite support, it was not significantly associated with the consumption of any of the 17 nutrients (p>0.05).

**Table 4.** Association of job strain and worksite support with daily intakes of 17 nutrients among employed men and women in Japan: Analysis of covariance (ANCOVA) of each nutrient intake on age, education, marital status, occupation, study site, job strain, and worksite support using baseline data of The Japan Work Stress and Health Cohort (JSTRESS) Study (1996-1998).

Dietary intake*	Job Strain^†^					Worksite Support^†^				
	
	1=low	2	3	4=high	p for trend	1=low	2	3	4=high	p for trend

	Men (n=15295)									
Total energy (kcal)	2176	2178	2180	2193	0.339	2166	2177	2168	2216	<0.001
Protein (g)	84.1	84.4	84.2	84.4	0.131	84.3	84.1	84.1	84.5	0.119
Fat (g)	57.6	58.0	58.1	58.3	<0.001	58.0	57.9	58.0	58.1	0.172
Carbohydrate (g)	315.9	315.1	315.0	315.2	0.252	314.8	315.5	315.5	315.4	0.616
Crude fiber (g)	4.58	4.60	4.57	4.58	0.675	4.58	4.56	4.56	4.63	0.001
Calcium (mg)	610.8	612.8	609.7	611.8	0.853	610.2	612.1	606.5	616.4	0.079
Retinol (*μ*g)	393.1	397.5	405.3	397.9	0.404	398.0	396.3	390.1	409.5	0.019
Carotene (*μ*g)	3882.7	3903.7	3865.4	3871.2	0.528	3852.7	3877.0	3849.8	3944.1	0.003
VA (IU)	3340.8	3365.5	3370.3	3361.4	0.495	3357.8	3347.7	3305.1	3428.5	0.003
VC (mg)	135.9	136.5	135.2	135.7	0.792	134.9	134.8	135.1	138.7	<0.001
VD (IU)	83.9	84.4	83.8	83.3	0.204	84.2	83.4	83.6	84.2	0.848
VE (mg)	7.65	7.70	7.68	7.71	0.035	7.70	7.65	7.67	7.73	0.036
Salt (g)	12.2	12.3	12.3	12.3	0.100	12.3	12.3	12.3	12.3	0.756
Cholesterol (mg)	287	289	289	290	0.015	288	287	289	290	0.039
Saturated fatty acid (g)	12.6	12.7	12.7	12.7	0.064	12.6	12.7	12.6	12.7	0.022
Polyunsaturated fatty acid (g)	13.4	13.4	13.4	13.5	0.012	13.4	13.4	13.5	13.4	0.785
Monounsaturated fatty acid (g)	17.0	17.1	17.1	17.2	0.001	17.1	17.0	17.1	17.1	0.151

	Women (n=2853)									
Total energy (kcal)	1991	2040	1996	2020	0.813	1991	1978	1996	2083	0.001
Protein (g)	91.0	91.0	91.6	91.2	0.408	90.7	91.4	91.0	91.7	0.045
Fat (g)	62.6	62.9	62.9	62.5	0.718	62.5	63.0	62.7	62.7	0.987
Carbohydrate (g)	314.4	313.3	313.2	313.7	0.538	314.0	312.8	314.0	313.8	0.770
Crude fiber (g)	5.40	5.30	5.40	5.40	0.906	5.30	5.40	5.30	5.40	0.070
Calcium (mg)	774.4	770.9	785.1	778.6	0.298	766.4	789.9	764.3	788.7	0.127
Retinol (*μ*g)	312.7	324.9	314.5	326.9	0.248	316.2	325.6	316.9	320.2	0.920
Carotene (*μ*g)	5374.5	5278.4	5493.1	5360.8	0.577	5262.4	5469.2	5282.2	5494.8	0.067
VA (IU)	3864.7	3864.4	3947.9	3926.7	0.209	3817.6	3987.4	3835.6	3965.3	0.121
VC (mg)	167.1	163.3	166.1	163.7	0.510	162.4	167.3	162.5	168.0	0.148
VD (IU)	96.5	95.9	98.8	96.2	0.708	96.5	96.3	97.1	97.4	0.447
VE (mg)	8.8	8.8	8.9	8.8	0.718	8.7	8.8	8.8	8.9	0.020
Salt (g)	13.8	13.9	14.0	14.0	0.086	13.8	14.0	14.0	13.9	0.484
Cholesterol (mg)	317	314	321	319	0.270	313	317	320	320	0.051
Saturated fatty acid (g)	13.7	13.7	13.7	13.7	0.883	13.6	13.9	13.6	13.8	0.812
Polyunsaturated fatty acid (g)	14.1	14.2	14.2	14.2	0.553	14.0	14.1	14.2	14.2	0.022
Monounsaturated fatty acid (g)	18.1	18.2	18.2	18.1	0.904	18.0	18.2	18.2	18.1	0.656

* : Nutrient intake (excluding total energy) was adjusted for total energy after a logarithm transformation of the crude values by the method described by Willett (1990). A log 10-transformed value for each dietary intake was used as a dependent variable in the ANCOVA. An antilog value of estimated least square means is shown.

†: Job strain and worksite support were classifed into quartiles. P for diff.: P for difference (d.f=3).

When the quartiles of job demands and job control, in place of those of job strain, were added to a similar ANCOVA, for men, job demands were significantly and positively associated with the total energy, fat, crude fiber, carotene, vitamins A and E intakes (p for trend <0.05). Also for men, job control was significantly and positively associated with total energy, crude fiber, calcium, carotene, and vitamin C (p for trend <0.05); job control was significantly and negatively associated with fat, and poly- and mono-saturated fatty acid intakes (p for trend <0.05). For women, job demands were significantly and positively associated with salt intake (p for trend <0.05).

These results were replicated by similar ANCOVAs among those who did not report any chronic medical condition (14,638 men and 2,793 women).

## DISCUSSION

Our study was the first to investigate the association between job strain, worksite social support and the intake of various nutrients. The study indicated that fat intake, and in particular poly- and monounsaturated fat intake, was significantly greater among employed men with a high degree of job strain, after controlling for age, education, marital status, occupation, and worksite support. Previous studies have also reported that job strain was positively associated with the intake of fat-rich foods among men in the US^5^ and with meat consumption in rural Japan when limited to non-agricultural occupations.^6^ However, in our study, the association between job strain and the intake of saturated fatty acid, which has been to be strongly associated with CHD,^8^ was only marginally significant in men. Our study indicated, rather, that job strain was significantly and positively associated with the intake of poly- and monounsaturated fatty acids that have some protective effects against CHD.^9^ Our findings do not support a hypothesis that fat intake may explain the link between job strain and CHD. However, the composition of fat associated with job strain may depend on the availability of fatty foods in a particular study area. A further replication study is therefore needed to address the association between job strain and the composition of fat in other countries.

Cholesterol intake was also greater among men under high job strain. Cholesterol is found in foods of animal origin (egg yolk, red meat, and dairy products). Another similar finding^6^ suggests that men under high job strain are more likely to prefer animal foods. However, the association of cholesterol intake with serum cholesterol concentration^30^ and CHD^31^ is usually moderate. It was reported that a change of 200mg/1000kcal in cholesterol consumption could explain the 30% of CHD incidence.^8^ The difference in average cholesterol intake per day between the lowest and highest quartiles of job strain was only 3.1 mg/day among men, which may explain the 0.1% of CHD incidence if the figure is applicable. Cholesterol intake may explain the association between job strain and CHD, but the degree seems limited.

Vitamin E intake was greater among men with high job strain. Although the high consumption (200-400 mg/day) of vitamin E has been reported to predict a lower risk of CHD,^32^ a later clinical trial failed to show this association.^33^ Because the observed difference was small, it does not seem that this level of change in vitamin E intake would have a clinically meaningful effect on CHD. Other vitamins were not associated with job strain in men or women, which is consistent with a previous observation.^7^ A previous observation of the association between job strain and little vegetable intake^6^ can probably be attributed to the fact that the study was conducted in a rural area where vegetable intake was greater and/or that the study sample was much older than those in the present study and a previous study.^7^

Among women, no significant association was noted between job strain and the consumption of any of the 17 nutrients observed in this study. A previous study reported that there was no significant association among women between job strain and the consumption of foods high in fat.^5^ The association of job strain with cardiovascular disease^34^ and general health^35^ was less clear in women than in men, and thus the link between job strain and nutrient intake may also be less clear in women than in men. This is also attributable to the smaller number of women in our sample.

Worksite support was found to be positively associated with daily intakes of crude fiber, retinol, carotene, and vitamins A, C, and E among men and with daily intakes of protein and vitamin E among women, although differences among the quartiles for nutrient intakes were small, particularly for crude fiber among men and vitamin E among men and women. This finding is in agreement with previous findings that general social interaction was positively associated with greater intakes of vegetables and fruit.^17^^,^^18^ Although the roles of these micronutrients and vitamins in preventing CHD are not clear,^8^ the consumption of fiber, protein,^9^ carotene,^32^ vitamin C,^35^ and vitamin E^32^ have shown to have some protection against CHD. Calcium intake was also found to be associated with a lower risk of hypertension.^36^ Therefore our findings may explain the association between worksite support and a lower risk of CHD^10^^-^^12^ to some extent. Workers who receive a high degree of worksite support may be more aware of the value of a healthy diet as a result of the information and emotional support that they receive from their fellow workers. Lower psychological strain among workers who receive greater worksite support may also make it easier for them to maintain their good health behaviors.

Among both men and women, total energy intake was greater for the groups with higher worksite support. Men who received a high degree of worksite support consumed more cholesterol and saturated fatty acid than did their counterparts. These findings are inconsistent with previous literature that reported a protective effect of worksite support on CHD.^10^^-^^12^ Worksite social support seems to be associated with both risk *and* protective factors for CHD. Workers who have good social relationships at their workplace may have more opportunities to eat high-calorie and fat-rich foods together at social gatherings with their supervisors and coworkers. Some of these associations between worksite support and nutrient intakes seemed non-linear, suggesting a more complex behind the influence of worksite support on dietary behaviors. Previous reports have suggested that peer support is sometimes associated with unhealthy lifestyles, such as smoking and drinking.^37^^-^^39^ More detailed dimensions of social interactions at workplaces should be considered in future studies in order to clarify this association between worksite support and diet.

The present findings on the association among job strain, worksite support, and diet may be specific to Japan because the availability of food is greatly different between Japan and most Western countries. Even if the diets for workers with a high degree of job strain were similar, e.g., a higher consumption of animal products, the consumption of nutrients in Japan and the US still might differ. In addition, a previous study conducted in rural Japan indicated that the association between job strain and diet was different between agricultural and non-agricultural workers.^6^ Our findings therefore need to be replicated among different occupations, between urban and rural areas, and across countries.

By applying a 31-item DHQ, we were able to obtain a more detailed picture of the association between the psychosocial work environment and diet. However, it should be noted that the DHQ is more suitable for ranking people according to nutritional intakes, rather than for estimating absolute values. Moreover, a random measurement error in the DHQ might attenuate a true association towards null. In such a case, we may have overlooked some important associations among job strain, worksite support, and nutrient intake. Furthermore, the assessment of the psychosocial work environment (job strain and worksite support) and diet was based on self-reported data, and therefore the tendency for individuals to provide affirmative responses may have confounded the findings. In addition, a cross-sectional design of the present study limits a causal interpretation. Some diets may also reduce psychological distress.^40^ In such cases, workers may report lower job strain and higher worksite support.

In conclusion, the present study found that job strain and worksite support were only weakly and inconsistently associated with nutritional intakes. It does not seem that changes in nutritional intakes explain the association between job strain or worksite support and CHD.

**Appendix.** Categorization of job strain, job demands, job control, and worksite support in men and women.

Group	Men (n=15,295)			Women (n=2,853)		
	
	(Range)	N	%	(Range)	N	%

	Job strain (job demands/job control ratio)						
1=low	(0.27-0.86)	3794	24.8	(0.37-0.92)	704	24.7
2	(0.86-0.96)	3838	25.1	(0.92-1.03)	708	24.8
3	(0.97-1.08)	3770	24.6	(1.04-1.21)	727	25.5
4=high	(1.08-3.37)	3893	25.4	(1.20-3.83)	714	25.0

	Job demands						
1=low	(12-29)	3858	25.2	(12-28)	786	27.5
2	(30-33)	4215	27.6	(29-31)	594	20.8
3	(34-36)	4631	30.3	(32-34)	807	28.3
4=high	(37-48)	2591	16.9	(35-48)	666	23.3

	Job control						
1=low	(24-62)	4568	29.9	(24-52)	726	25.4
2	(63-68)	3655	23.9	(53-60)	826	29.0
3	(69-74)	3752	24.5	(61-66)	718	25.2
4=high	(75-96)	3320	21.7	(67-96)	583	20.4

	Worksite support (supervisor support + coworker support)						
1=low	(8-19)	2881	18.8	(8-19)	684	24.0
2	(20-22)	4309	28.2	(20-21)	507	17.8
3	(23)	2682	17.5	(22-23)	857	30.1
4=high	(24-32)	5423	35.5	(24-32)	805	28.2
